# The use of informativity in the development of robust viromics-based examinations

**DOI:** 10.7717/peerj.3281

**Published:** 2017-05-02

**Authors:** Siobhan C. Watkins, Catherine Putonti

**Affiliations:** 1 Biology Department, New Mexico Institute of Mining and Technology, Socorro, NM, United States of America; 2 Department of Biology, Loyola University of Chicago, Chicago, IL, United States of America; 3 Department of Computer Science, Loyola University of Chicago, Chicago, IL, United States of America; 4 Bioinformatics Program, Loyola University of Chicago, Chicago, IL, United States of America; 5 Department of Microbiology and Immunology, Loyola University of Chicago, Maywood, IL, United States of America

**Keywords:** Virome, Metagenomics, Bacteriophage, Viral community

## Abstract

Metagenomics-based studies have provided insight into many of the complex microbial communities responsible for maintaining life on this planet. Sequencing efforts often uncover novel genetic content; this is most evident for phage communities, in which upwards of 90% of all sequences exhibit no similarity to any sequence in current data repositories. For the small fraction that can be identified, the top BLAST hit is generally posited as being representative of a viral taxon present in the sample of origin. Homology-based classification, however, can be misleading as sequence repositories capture but a small fraction of phage diversity. Furthermore, lateral gene transfer is pervasive within phage communities. As such, the presence of a particular gene may not be indicative of the presence of a particular viral species. Rather, it is just that: an indication of the presence of a specific gene. To circumvent this limitation, we have developed a new method for the analysis of viral metagenomic datasets. BLAST hits are weighted, integrating the sequence identity and length of alignments as well as a taxonomic signal, such that each gene is evaluated with respect to its information content. Through this quantifiable metric, predictions of viral community structure can be made with confidence. As a proof-of-concept, the approach presented here was implemented and applied to seven freshwater viral metagenomes. While providing a robust method for evaluating viral metagenomic data, the tool is versatile and can easily be customized to investigations of any environment or biome.

## Background

Bacterial viruses (bacteriophages) play a crucial role in shaping microbial populations and processes on a global scale. They shape community structure via mediation of mortality and drive diversity as agents of genetic mobility (Wilhelm & Suttle, 1999; Canchaya et al., 2003; Berdjeb et al., 2011; Clokie et al., 2011; Winget et al., 2011; Willner et al., 2012; Brum et al., 2016; Manrique et al., 2016), and their impact has been described at higher trophic levels (Rohwer & Thurber, 2009; Jover et al., 2014). Despite being the most ubiquitous and abundant biological entity on the planet, only a comparatively small fraction of phage genomes has been sequenced (Klumpp, Fouts & Sozhamannan, 2012). Nevertheless, from this small and imprecise representation of phage diversity we have uncovered a great deal about their genomes: they span a remarkable degree of genetic diversity and often have highly mosaic genome architectures (Hatfull, 2008; Hatfull, 2015). The majority of phage genes, however, are unfamiliar to us, their function unknown (Hatfull, 2008; Sharon et al., 2011). Nevertheless, as is true of all aspects of microbial diversity in the environment, the significance of the work performed to date does not negate how much there is left to discover.

Numerous studies of phage communities spanning a wide variety of environments, from the human gut (Minot et al., 2013) to terrestrial hot springs (Gudbergsdóttir et al., 2015), have repeatedly found that we are underestimating the genetic diversity within phage populations (Dinsdale et al., 2008; Halary et al., 2010; Hurwitz & Sullivan, 2013; Paez-Espino et al., 2016). Conserved taxonomic “gene signature” sequences (e.g., g20 (Short & Suttle, 2005) and g23 (Filée, Tétart & Krisch, 2005)) are far from comprehensive (Adriaenssens & Cowan, 2014); and there are likely groups in nature that do not contain a single signature gene identified within existing clades. Thus, whole genome sequencing (WGS) is widely considered to be the most representative method for exploring viral diversity in the environment. Bioinformatic approaches for analyzing viral metagenomes largely mirror those used for the study of bacterial and archaeal populations: reads or contigs are compared to known, characterized sequences within public data repositories. While comparisons can be made to, e.g., all viral genome sequences, another option is direct comparison to Prokaryotic Virus Orthologous Groups (pVOGs, formerly called Phage Orthologous Groups, POGs) (Kristensen et al., 2010; Kristensen et al., 2013; Grazziotin, Koonin & Kristensen, 2017), including 57 taxon-specific “signature” sequences (Kristensen et al., 2013). This approach has been employed frequently (e.g., Kristensen et al., 2010; Waller et al., 2014; Jeffries et al., 2015; Laffy et al., 2016) and these taxon-specific signatures include genes that are not found in genomes of other viral taxa. But the diversity of phages is severely undersampled, and therefore it is not surprising then that only a small fraction of sequences from viral metagenomic surveys exhibit any homology to extant databases or these signature sequences (Hurwitz & Sullivan, 2013; Bruder et al., 2016; Paez-Espino et al., 2016).

For the few viral species that can be identified, typically via BLAST searches against complete viral genomes or the aforementioned POG/pVOG sequences, the best hit is often regarded as being representative of the viral taxon containing the homologous region (particularly if the hit is to one of the taxon-specific signatures). This approach is employed by many metagenomics-based studies, analytical tools, and metrics (e.g., Wommack et al., 2012; Huson & Weber, 2013; Roux et al., 2014; Aziz et al., 2015; Keegan, Glass & Meyer, 2016). Homology-based classifications, however, can be misleading due to two factors. Firstly, phage genomes available in public repositories: (a) capture but a small fraction of the viral diversity on Earth, (b) represent phages with hosts amicable to growth under laboratory conditions, and (c) phage groups have very biased sampling rates (e.g., the heavily sampled Mycobacteriophage vs. the less-sampled phages of *Burkholderia*) (Bruder et al., 2016). Secondly, lateral gene transfer (LGT) is pervasive within phages communities. There is an abundance of evidence of LGT between phages with similar host ranges, between phages within the same environment, and between phages and their hosts (e.g., Mann et al., 2003; Brussow, Canchaya & Hardt, 2004; Lindell et al., 2005; Lima-Mendez et al., 2008; Thompson et al., 2011; Gao, Gui & Zhang, 2012).

Here, we introduce a rigorous method for classifying viromes. Genes exhibiting homology to characterized sequences are weighted based upon their *informativity*—a new metric for describing viral community structure. This metric provides a means for distinguishing (and qualifying this distinction) between the presence/absence of a particular taxonomical group and genic content. Thus, it is possible to distinguish between genes indicative of a particular taxa and those that are frequently exchanged within viral communities. In addition to presenting the method, we have tested its robustness through the analysis of all individual genera of tailed bacteriophages (order: *Caudovirales*). As a proof-of-concept, we examined seven publicly available freshwater DNA metagenomic datasets.

### Materials and Methods

### Development of the informativity metric

#### Establishing a taxonomic signal threshold

To ascertain the presence/absence of a specific taxon within a metagenome, we suggest a threshold to differentiate between informative and uninformative hits. The taxonomic signal threshold *T* is determined through a two-step process prior to evaluation of the metagenomic data. In the first step, each annotated coding region for a given taxon of interest is compared to all annotated sequences within the genome(s) of a known relative. Thus, each coding region’s sequence *x* (*x* ∈ *X*, where *X* is the set of sequences for all coding regions annotated within the genome of the taxon of interest) is compared to each coding region’s sequence *g* (*g* ∈ *G*, where *G* is the set of sequences for all coding regions annotated within the genome of a known relative). The use of a known relative genome(s) establishes if and how conserved the coding region is between known, related strains/species. Where sequence homology is detected, the sequence identity and query coverage of the match is recorded: *S*_1_ and *Q*_1_, respectively.

**Figure 1:**
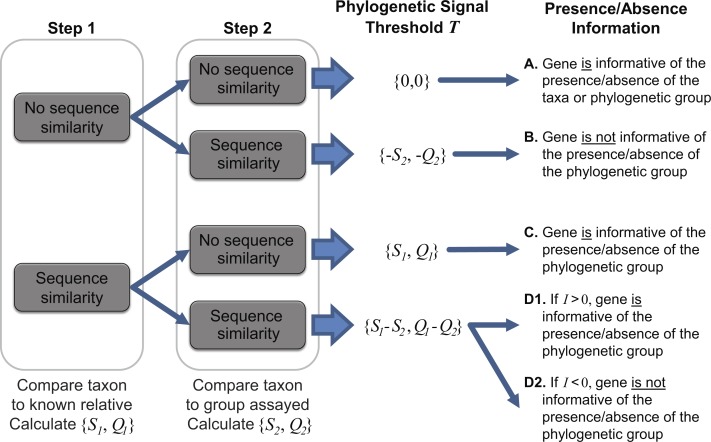
Two-step process for determining the taxonomic signal threshold *T* and the information which can be gained regarding the presence/absence of a taxon’s phylogenetic group. *S*_1_ and *S*_2_ represent the sequence identity of homologies identified in step 1 and 2, respectively. Likewise, *Q*_1_ and *Q*_2_ refer to the query coverage of the match detected in step 1 and 2, respectively.

In the second step, each coding region’s sequence is compared again, this time to the sequences for all annotated coding regions for the group assayed by the metagenome (e.g., all phages, viruses, bacteria, archaea, etc.), however, those belonging to the taxonomic group containing the taxon of interest and the known relative considered in step one are omitted. Many hits may be recorded for a particular gene *x*. Thus the best hit, the highest scoring hit both with respect to the sequence identity and the query coverage of the match, is selected; *S*_2_ and *Q*_2_ denote this best match’s sequence identity and query coverage, respectively. A taxonomic signal threshold *T* is defined as *T* = {*S*_1_−*S*_2_, *Q*_1_−*Q*_2_} where the subscripts *1* and *2* represent the sequence identity and query coverage of the match detected from steps one and two, respectively. Figure 1 illustrates the two-step process and the *T* values produced.

It is important to note that the taxonomic group used for comparison is user defined. For instance, in order to ascertain if a gene can be used to distinguish between the presence/absence of a particular species, one may consider the taxonomic group to be inclusive only of strains of the species. Therefore, in this case, the most distant relative belonging to the taxonomic group in step one would be the closest related species. If a more distant relative, say the most distantly related species of the same genus, were to be investigated, then the taxonomic signal threshold *T* would serve as a means to distinguish between the presence/absence of a subset of the species (inclusive of the taxon of interest) within the genus. This flexibility enables the researcher to define and control the granularity of his/her analyses. If a particular taxa of interest lacks available genomes capturing the phylogenetic diversity of the species (or genus or subfamily, etc.), a more distant relative can be selected. In addition to the intended purpose of establishing the taxonomic signal threshold, the two-step process can provide insight into putative horizontally acquired elements and gene loss events, e.g., instances in which the gene did not include a homolog in the most distant relative but did exhibit sequence similarity to a gene within the genome of another taxonomic group.

#### Using informativity to ascertain confidence in taxonomical calls

As indicated in Fig. 1, when *T* is greater than zero (outcomes C and D1), the presence of a specific gene can provide insight. Operational Taxonomic Unit (OTU) calls are informed by this threshold to decipher BLAST analyses of metagenomic datasets as some hits may be to genes which are conserved and thus poor indicators if a species/taxa is present or absent. For a given hit within a metagenomic dataset, the sequence identity and query coverage, *S*_*H*_ and *Q*_*H*_ respectively, is assessed relative to the taxonomic signal threshold *T* for the gene producing the match. Genes in which *T* <0 have already been classified as uninformative (Fig. 1). Hits which fall below the gene’s threshold, {*S*_*H*,_ *Q*_*H*_} < *T*, are also classified as uninformative, while hits which are above the threshold are considered informative. The informativity *I* of each hit is quantified based upon deviation from this threshold *T* such that *I* = {*S*_*H*_, *Q*_*H*_}-*T*. *I* can range from 0 (equivalent to the threshold *T*) to 100 (*T* = {0,0}, *S*_*H*_ = *Q*_*H*_ = 100%). Thus, genes with a high value of *I* are strong indicators of the presence of the gene from the taxon of interest (or a closely related strain/species) within a metagenomic dataset.

Taking into consideration the number of informative genes detected within a metagenomic sample and their individual *I* values, one can then quantify with confidence the likelihood of the presence/absence of the taxon of interest. For example, consider the case in which a novel species, *n*, within a genus is represented within a metagenome. It shares homology with other genomes for the genus. For the sake of simplicity assume there are two other genomes for the genus: *a* and *b*. The novel species *n*’s genome contains a subset of genes that are more similar to informative genes in *a*’s genomes and some genes that are more similar to informative genes in *b*’s genome. One can use the informativity values calculated for the genes of *n* to provide a confidence value in calling the contig a representative of *a* and/or *b*. Furthermore, rather than simply assign the contig as a representative of *a* or *b* or simply a member of a particular genus based upon a single signature gene, the informativity metric can provide insight into the evolutionary history of this novel species and the taxa.

### Implementation

The method for assessing the informativity of viromic hits was implemented using a series of BLAST databases and BLAST searches. A collection of all coding regions (nucleotide sequences) for the taxon of interest (*X*) and all genes (amino acid sequences) annotated within the genome of the selected relative (*G*) are supplied by the user. A local BLAST database is created for *G,* and the genes belonging to *X* are queried against the local database via blastx. The sequence identity and query coverage of the match detected for the best hit for each gene is then parsed from the BLAST results quantifying each gene’s *S*_1_ and *Q*_1_ values. Next, a BLAST database is created for the annotated coding regions (amino acid sequences) provided for step 2 of this method (set *Z*), again supplied by the user. Each of the genes for the taxon of interest *X* is queried against this second local database via blastx; the results are again parsed for each gene’s *S*_2_ and *Q*_2_ values so that the taxonomic signal threshold *T* can be calculated.

A metagenomic dataset can next be evaluated, comparing each read or contig against a collection of annotated gene sequences. To accommodate the variation between characterized sequences in databases and environmental samples, contigs are translated—generating all six open reading frames—and a protein database representative of the metagenomic dataset is produced. Each BLAST hit is next assessed with respect to its scores {*S*_*H*_, *Q*_*H*_} relative to that of the gene’s threshold *T*. For each gene in the genome of interest *X*, the values for *S*_1_, *Q*_1_, *S*_2_, *Q*_2_, *S*_*H*_, and *Q*_*H*_ are written to file. The user can then evaluate the likelihood of a particular taxon’s or taxonomic group’s presence within the metagenomic sample based upon the *I* values for informative genes. Note that for the analyses presented here we have weighted *S* and *Q* values equally in the calculation of *T*; the two values are, however, reported separately such that users can select their own weighting of the contributions of sequence identity and query coverage.

The described process has been automated via functionality developed in C++ (available for both Windows and Unix OS). Users must supply or specify the FASTA format files for the taxon of interest (*X*), the known relative (*G*), and the group assayed (less the taxonomic group of interest) (*Z*). If metagenomic comparisons are to be conducted, as this is optional in the current implementation, the user must also supply the metagenomic dataset. The code has been designed for both ease of use, speed, and flexibility, such that analyses can be tailored to the environmental niche and/or hypothesis under investigation. Most importantly, this is a light-weight solution which can be integrated into the standard method of viral metagenomic analyses. Source code, documentation, and sample data are publicly available at https://github.com/putonti/informativity.

### Datasets examined

#### Viral gene and genome datasets

Sequence data were retrieved from NCBI GenBank (NCBI Resource Coordinators, 2017) (collected August 2016). Datasets for 70 taxonomical groups within *Caudovirales* were retrieved (Table S1); searches were conducted in NCBI for protein sequences through an advanced search query: PHG[Division] AND txidXXXXX[Organism] (where the X’s refer to the NCBI Taxonomy Browser’s Taxonomy ID number). Note, this only collects phages that have been annotated to the taxon (i.e., their genome has been annotated with the Taxonomy ID). From these queries, 70 sets of genome sequences were retrieved. Sixty-four individual genera were selected. The other six sets consist of sequences for species belonging to the same subfamily. *Caudovirales* taxa were selected as they are the largest and best characterized phage genomes currently available (Salmond & Fineran, 2015). In addition, phages classified within other orders were retrieved with the following query: (PHG[Division] NOT txid28883[Organism]); Taxonomy ID 28883 is the unique identifier for *Caudovirales*. The results of this query include all phages belonging to other orders (1,003 phage strains in total). For each *Caudovirales* taxonomical group, the type species’ genome was retrieved, again from NCBI. The type species was determined by referring to the 2015 release by the International Committee on Taxonomy of Viruses (ICTV) (http://www.ictvonline.org). The type species for each *Caudovirales* taxonomical group is listed in Table S1 .

In our proof-of-concept analyses of the *Pbunavirus Pseudomonas phage PB1*, we verified the taxonomic classification of Pbunaviruses. Genomes exhibiting significant homology (>50% of coding regions) to PB1 that were not assigned to the *Pbunavirus* Taxonomy ID were further investigated. The complete sequence of the genome in question was aligned via the blastn algorithm through the NCBI BLAST site. Alignments with a query coverage and percent identity greater than 50% were identified and the literature was referenced to correctly assign the taxonomic classification. Additional *Pbunavirus* strains were identified from the “unclassified *Myoviridae*” following this above method. These genomes were thus reannotated for our subsequent analysis of viral metagenomic datasets as *Pbunavirus*. (See Table S2 for a list of the genomes classified here as Pbunaviruses.) Pbunaviruses were selected for this proof-of-concept work given our prior isolation and identification of *Pseudomonas phage PB1* in the freshwaters of Lake Michigan (Malki et al., 2015).

#### Viral metagenomic analyses

SRA records were collected from the NCBI SRA database (http://www.ncbi.nlm.nih.gov/sra). Table S3 lists all of the datasets included in the proof-of-concept study. Each SRA record (line listed in the Table S3) was considered as an individual sample with two exceptions. Two samples are aggregates of more than one SRA record, both belonging to Virome IV, as they were combined in the downloadable file from the SRA database. Our dataset includes 56 individual samples. These samples were chosen as they target DNA viruses in similar environments (freshwater). Furthermore, they are rather well documented in the literature. Each individual sample was next assembled using Velvet (Zerbino & Birney, 2008) with a hash size of 31; default values were used for all other parameters. Each sample was thus uniformly prepared for analysis.

The amino acid and nucleotide sequences for *Pseudomonas phage PB1* (type strain for the *Pbunavirus* genus; Accession Number: NC_011810) and *Burkholderia phage BcepF1* (Accession Number: NC_009015) were downloaded from NCBI for comparison with the virome datasets. All phage nucleotide sequences (omitting those belonging to the *Pbunavirus*) were also retrieved via the advanced search query: PHG[Division] NOT txid1198980[Organism] (where the Taxonomy ID listed is that for *Pbunavirus*). In total over 500000 individual records were retrieved, including partial and complete sequences. The informativity values are visualized in later figures as heatmaps that were produced in Excel.

## Results and Discussion

### Identifying informative genes

**Figure 2:**
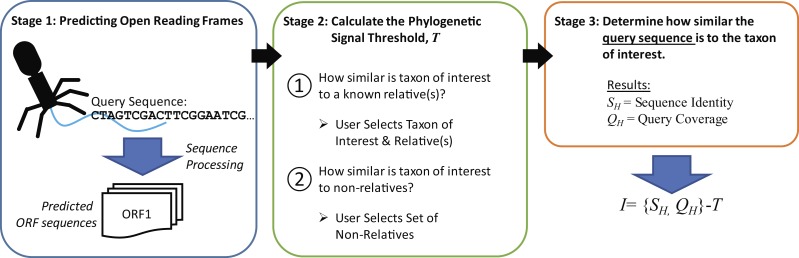
Process for calculating informativity. In Stage 1, users supply their assembled contigs which are processed, predicting ORFs. Users must supply at minimum two sequences for the taxon of interest (preferably spanning the diversity of sequences within the taxon) and sequences of ’non-relatives’ for the calculation of the phylogenetic signal threshold *T* in Stage 2. Each gene’s informativity is calculated in Stage 3.

The new metric described here, Informativity or *I*, provides a quantifiable means of identifying if a particular taxonomical group is present/absent within a sequenced community. Developed specifically for the detection of viral sequences in complex community metagenomic data sets, *I* captures the likelihood of a sequence belonging to taxa. Described in greater detail within the Methods, Fig. 2 provides an overview of how informative genes are identified. Users must supply the query sequence(s) (likely a contig or set of contigs from a sequenced community), at least two representative sequences for a taxon of interest, and lastly a set of sequences representative of ‘non-relatives’ (sequences belonging to other taxa of, e.g., viruses). The taxon of interest can be, e.g., a species, a genus, or a subfamily.

### Informative genes for *Caudovirales* taxa

All protein coding sequences were collected for species belonging to 70 tailed-virus (*Caudovirales*) taxa identified by NCBI Taxonomy (see Methods). Using the ICTV type species as a representative of the taxa, each gene sequence (*x*) of the type species’ genome (*X*) was compared to all other gene sequences for species of the same taxa. For each gene, the sequence identity *S*_1_ and query coverage of the match *Q*_1_ for the most dissimilar homologous gene sequence within the taxa is calculated. This captures the sequence variation for the gene within the species of the taxon. Thus, the *S*_1_ and *Q*_1_ scores for one gene *x*_*i*_ may be from homology detected in one species of the taxa, while the scores for another gene *x*_*j*_ may be to a homolog in another species’ genome. If the gene is unique to the type species’ genome, then *S*_1_ = *Q*_1_ = 0. The sequence identity, *S*_2_, and query coverage, *Q*_2_, scores were next calculated for each gene in the type species’ genome; each gene was compared to: (1) genes belonging to species classified within other genera within the order *Caudovirales* and (2) genes belonging to species of other taxonomic orders. In contrast to the *S*_1_ and *Q*_1_ scores, the *S*_2_ and *Q*_2_ scores are for the best hit or the most similar homolog found. Using these two values, the taxonomic signal threshold *T* can be calculated (see ‘Methods’). This threshold value signifies how reliable the particular gene is as an indicator of the presence/absence of the species. Genes which are found in multiple species and taxa would thus have a low threshold value *T* and perform poorly as an indicator of the taxon.

**Figure 3:**
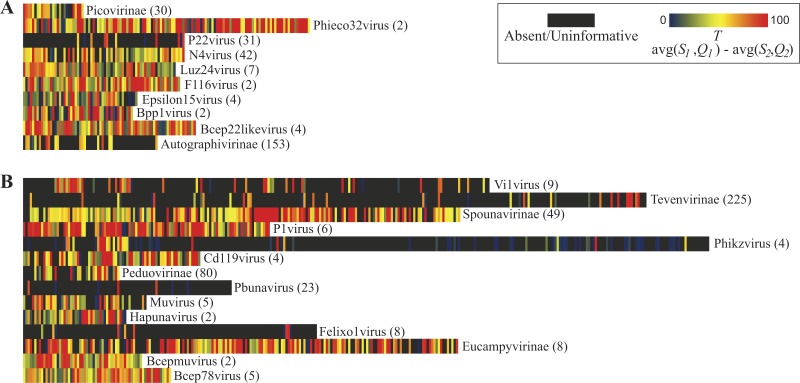
Taxonomic signal threshold value *T* for each gene within phage type species of taxonomic groups belonging to the family *Podoviridae* and *Myoviridae*. For each taxonomical group belonging to the viral family **(A)** *Podoviridae* and **(B)** *Myoviridae,* the number of genome sequences examined (including the type strain) is indicated in parentheses.

Figure 3 illustrates the thresholds for *Myoviridae* and *Podoviridae* type species; *Siphoviridae* is included in Fig. S1. (Type species names and accession numbers as well as scores are listed in Table S1). In these maps, each gene’s taxonomic signal threshold is shown; dark gray boxes indicate uninformative genes; these uninformative genes either exhibit greater homology to species belonging to other phage taxa or lack homology to other representative genomes of the taxon of interest (i.e., are present only within the type species’ genome). Also listed for each taxon is the number of genome sequences included in the comparisons. Those taxonomical groups with more phylogenetic diversity represented within available genome sequences tend to have less informative genes. This is quite prominent when evaluating the 10 *Podoviridae* taxon: the well sampled subfamily of *Autographivirinae* species have significantly less informative genes than the undersampled *Podoviridae* genera of, e.g., F116virus and Bpp1virus. It is important to note, however, that taking into consideration numerous genome sequences does not necessarily mean that the phylogenetic diversity of the taxon was examined. In contrast to classifying unknown sequences by a single marker, the informativity metric provides a multiple gene marker strategy. Thus, taxonomical ‘calls’ for a sequence can be made with greater confidence by reporting the aggregate of informative markers found, not just the presence/absence of a single gene.

### Targeting specific phages in environmental samples

**Figure 4:**
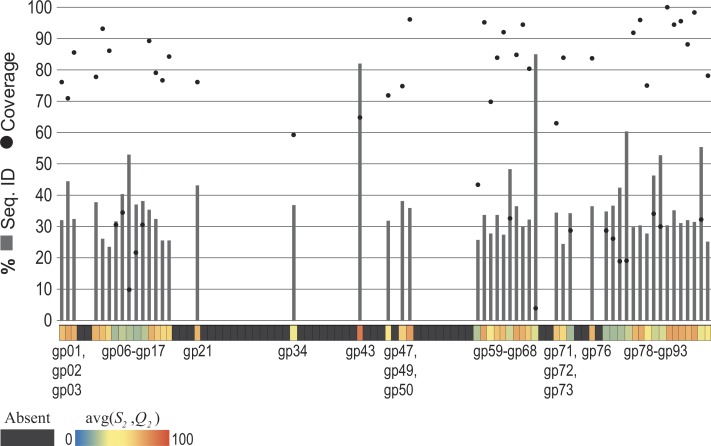
Observed similarity of each *Pseudomonas phage PB1* gene to phage genes within other (non-*Pbunavirus*) taxa. The percent sequence identity (bars) and percent query coverage (circles) values for the best hit for each gene is shown as is the average of these two percentages within the heatmap along the *x*-axis. Genes which do not show homology to non-Pbunaviruses are indicated as dark gray boxes within the heatmap.

The *Pseudomonas phage PB1* was selected for examination. Each gene annotated for the PB1 genome (Accession Number: NC_011810) (Ceyssens et al., 2009) was compared first to the set of genes for the most distant relative of PB1 within its genus *Pbunavirus* (previously called *Pbunalikevirus*), *Burkholderia phage BcepF1* (Accession Number: NC_009015). For each gene the *S*_1_ and *Q*_1_ values were computed. Next, all 93 annotated PB1 genes were compared to all genes from phage species—other than those classified as Pbunaviruses (see ‘Methods’). Homologous sequences were identified, the *S*_2_ and *Q*_2_ values. The similarity observed (the *S*_2_ and *Q*_2_ values) for each of the PB1 genes is shown in the heatmap of Fig. 4. Several PB1 gene sequences (as indicated by the color scale) exhibited sequence homology to genes within phage genomes of other taxa. Dark gray blocks in the heatmap signify that no homologs were detected. The upper chart in Fig. 4 details the percent sequence identity (bars) and percent query coverage (circles) values observed for the best hits to GenBank records. PB1 genes with homologies to other phage taxa include conserved genes (e.g., gp47 = tail fiber component and gp50 = DNA ligase), amongst other conserved “hypothetical proteins”.

**Table 1:** Freshwater DNA viral metagenomic studies retrieved from NCBI’s SRA database.

Virome	Environmental niche	Number of samples	Sequencing technology	Mbp total	Reference
I	Lake Michigan nearshore	40	Illumina	6,909	Watkins et al. (2015), Sible et al. (2015)
II	Lake Bourget	2	454	698	Roux et al. (2012)
III	Kent SeaTech tilapia pond	3	454	47	Dinsdale et al. (2008)
IV	Lake Limnopolar	2	454	18	López-Bueno et al. (2009)
V	Reclaimed water samples	6	454	364	Rosario et al. (2009)
VI	Lake Ontario	3	454	223	n/a
VII	Feitsui Reservoir	5	454	86	Tseng et al. (2013)

**Figure 5:**
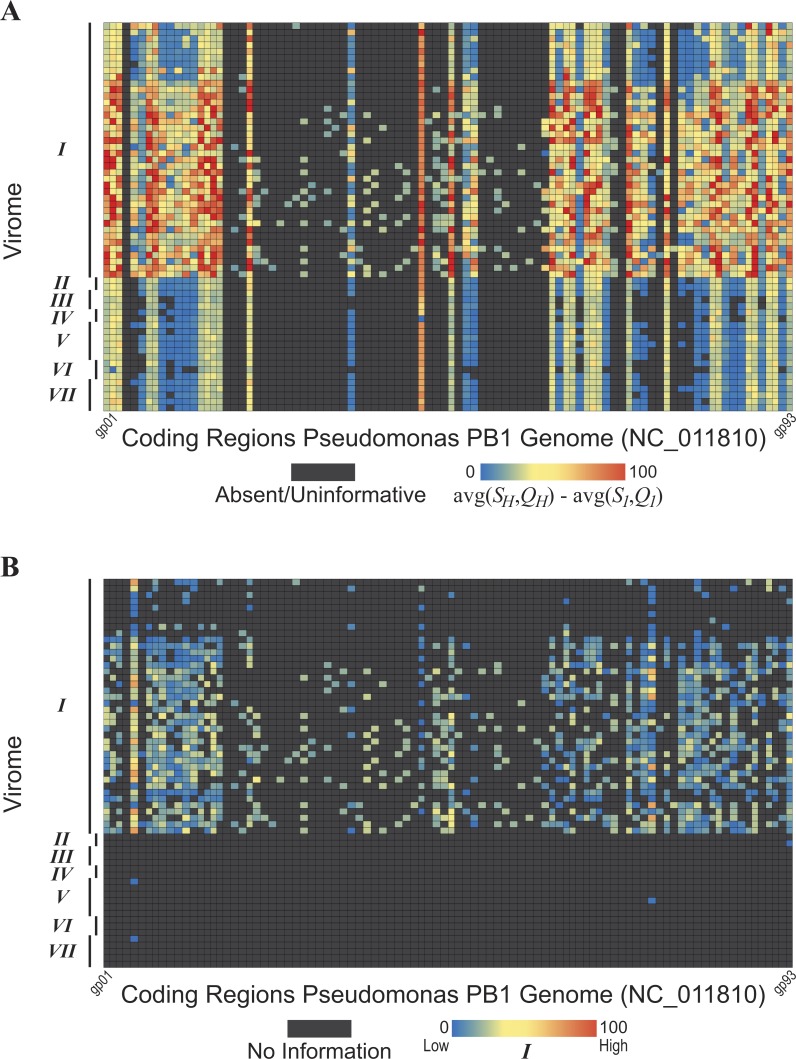
Evidence of *Pseudomonas phage PB1* genes within seven freshwater DNA viromes The seven viromes correspond to those listed in Table 1. (A) Hits (*S*_*H*_ and *Q*_*H*_) to PB1 genes within viromes. As shown by the color scale, some hits to PB1 genes are better (in terms of sequence identity and query coverage) than homologies observed with the distant *Pbunavirus Burkholderia phage BcepF1*. (B) Hits are qualified relative to the taxonomic signal threshold *T* calculated for PB1 genes.

The methodology developed here was then applied to seven freshwater DNA viromes (Table 1); a list of the SRA datasets from each study is provided in Table S3. Each of the 56 samples examined was first assembled (see ‘Methods’ for details). The PB1 coding regions were then compared to the 56 collections of contigs. The heatmap shown in Figure 5A graphically represents these results; each row represents a single sample (Methods). Again, each gene’s best hit within each virome’s sample was qualified (colored) with respect to its conservation amongst the Pbunaviruses, the gene’s *S*_1_ and *Q*_1_ value. Nevertheless, not all genes provide an equal signal as to the presence or absence of PB1 within the sample: some serve as better markers. As shown in Fig. 4, there are several “non-*Pbunavirus*” species which contain homologs to PB1 genes. Thus, the informativity *I* of each BLAST hit within the seven viromes was calculated. In doing so, individual genes that provide a strong signal for the *Pseudomonas phage PB1* can readily be identified. Figure 5B represents the results of this computation, in which each hit to a PB1 gene is now assessed in light of the taxonomic signal threshold *T*.

In an effort to assess the strength of the metric presented here, we evaluated the raw BLAST results of the datasets and a BLAST score-based analysis. The BLAST results of Viromes II, IV, V, and VII are publicly available through the web service MetaVir (Roux et al., 2014). Nine of the samples from Virome I are also available through MetaVir. It is important to note that in contrast to the uniform method in which the viral metagenomes were preprocessed here (see ‘Methods’), the sequences submitted to MetaVir, or comparable online resources, may be assembled or raw sequences. Furthermore, MetaVir conducts BLAST comparisons against the RefSeq viral database (O’Leary et al., 2016), whereas here we have included all partial and complete phage sequences from GenBank. Nevertheless, hits to the *Pbunavirus* (Table S2) genomes were identified in all five MetaVir datasets; the Lake Michigan and Lake Bourget samples (nine samples from Virome I and both samples from Virome II, respectively) produced the most hits in MetaVir to the *Pbunavirus* genomes (hundreds to thousands), many which were the best hits identified. Hits from MetaVir metagenomic samples, including Viromes I, II, IV, V, VII and additional sampling sites not included in our proof-of-concept work, to the *Pseudomonas phage PB1* genome are shown in Fig. S2.

Virome I, the Lake Michigan viral metagenomes generated by our group (Watkins et al., 2015; Sible et al., 2015), includes many informative genes (Fig. 5B) indicative of the presence of a *Pbunavirus* similar to PB1. Thus, with confidence, one can predict its presence within this sample. Viromes II, V, and VII contain far fewer hits to informative genes (one, two, and one PB1 genes respectively). Furthermore, their informativity scores are low, {*S*_*H*_*, Q*_*H*_}≈ *T*. This would suggest that PB1 (or a close relative) is not present within the sample: rather a homolog of the gene is present, within an uncharacterized species. As viral sequence databases expand through the isolation and characterization of additional viruses, the threshold *T* is likely to change thus providing greater confidence in the evaluation of BLAST hits for OTU calling.

## Conclusions

The method presented here, for extrapolating the presence/absence of microbial taxa, is robust and versatile. By scrutinizing a set of informative genes, the effects of lateral gene transfer and incomplete, sparse databases are reduced. Furthermore, as new genome sequences are released, the informativity metric can be easily updated. Specifically, the proof-of-concept investigation of seven freshwater virome datasets can be applied to identify novel strains and species of phages with confidence and thus easily mine large datasets for specific taxa of interest. Many of the cellular constituents of the human microbiome are undergoing examination, and exploration of human viromes is certainly the next frontier (Abeles & Pride, 2014; Ogilvie & Jones, 2015; Handley, 2016; Manrique et al., 2016; Zou et al., 2016). These studies have already discovered novel phage species (Dutilh et al., 2014; Malki et al., 2016) and will undoubtedly continue to increase our understanding of phage diversity. Nevertheless, improved bioinformatic tools for mining sequences representative of complex viral communities, coupled with further physical isolation and characterization of viral species have the potential to greatly expand our knowledge of the viral diversity on Earth.

## Supplemental Information

10.7717/peerj.3281/supp-1 Figure S1 Taxonomic signal threshold value *T* for each gene within phage type species of genera belonging to the family *Siphoviridae* For each genus, the number of genome sequences examined (including the type strain) is indicated in parentheses.

10.7717/peerj.3281/supp-2 Figure S2 Hits from MetaVir metagenomic samples to the *Pseudomonas phage PB1* genome

10.7717/peerj.3281/supp-3 Table S1 Type species name and accession number for each genus of *Caudovirales* For each coding region within each type species’ genome, the *S*_1_, *Q*_1_, *S*_2_, and *Q*_2_ scores are reported.

10.7717/peerj.3281/supp-4 Table S2 Genomes classified as Pbunaviruses in this study through genome comparison The taxonomy as listed in NCBI includes *Pbunavirus* and unclassified *Myoviridae*.

10.7717/peerj.3281/supp-5 Table S3 Freshwater SRA datasets examined SRA datasets included in the proof-of-concept investigation for *Pseudomonas phage PB1* viruses. Each row listed was considered an individual sample.

## Additional Information and Declarations

### Data Availability

The following information was supplied regarding data availability: GitHub: https://github.com/putonti/informativity.
