# Supplementary figures and images for: The use of informativity in the development of robust viromics-based examinations
Source: PeerJ. 2017 May 2;5:e3281. doi: 10.7717/peerj.3281 (PMC5417064; doi:10.7717/peerj.3281)

NC\_011810

Number of predicted genes

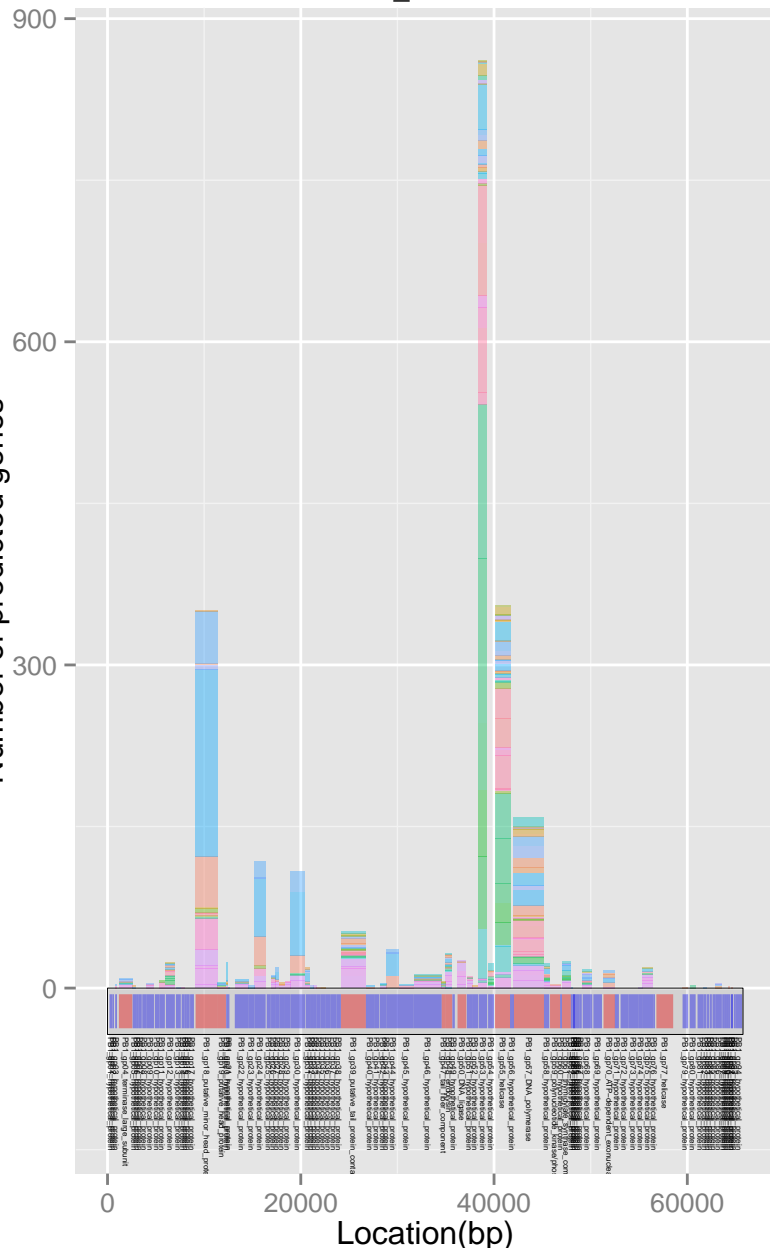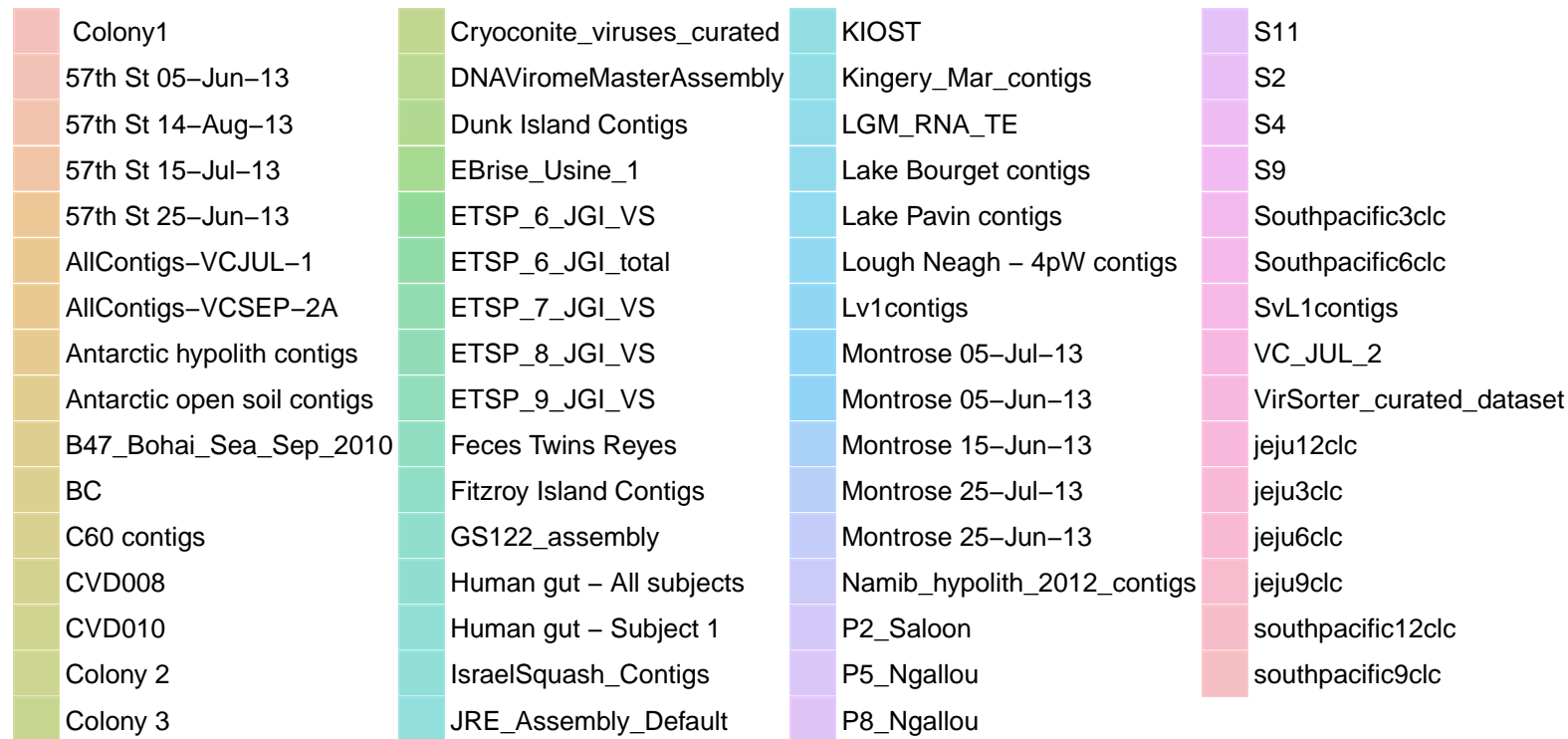

Supplement: Figure S2 [file peerj-05-3281-s002.pdf]
