# Supplementary material for: The use of informativity in the development of robust viromics-based examinations
Source: PeerJ. 2017 May 2;5:e3281. doi: 10.7717/peerj.3281 (PMC5417064; doi:10.7717/peerj.3281)
Supplement: Table S2 — The taxonomy as listed in NCBI includes Pbunavirus and unclassified Myoviridae. [file peerj-05-3281-s004.docx]

**Table S2.** Genomes classified as *Pbunaviruses* in this study through genome comparison. The taxonomy as listed in NCBI includes *Pbunavirus* and unclassified *Myoviridae*.

| **Phage strain** | **Genome size** | **NCBI Assigned Taxonomy** | **GenBank Accession No.** |
| --- | --- | --- | --- |
| PB1 | 65764 | *Pbunavirus* | NC_011810 |
| SN | 66390 | *Pbunavirus* | NC_011756 |
| 14-1 | 66238 | *Pbunavirus* | NC_011703 |
| LMA2 | 66530 | *Pbunavirus* | NC_011166 |
| LBL3 | 64427 | *Pbunavirus* | NC_011165 |
| F8 | 66015 | *Pbunavirus* | NC_007810 |
| BcepF1 | 72415 | *Pbunavirus* | NC_009015 |
| PaMx13 | 66450 | *Pbunavirus* | JQ067083 |
| pp DL52 | 65867 | *Pbunavirus* | KR054028 |
| pp SPM-1 | 65729 | *Pbunavirus* | NC_023596 |
| pp vB_PaeM_C1-14_Ab28 | 66181 | unclassified *Myoviridae* | NC_026600 |
| pp DL60 | 66103 | unclassified *Myoviridae* | KR054030 |
| pp KPP12 | 64144 | unclassified *Myoviridae* | NC_019935 |
| pp NH-4 | 66116 | unclassified *Myoviridae* | NC_019451 |
| pp vB_PaeM_PAO1_Ab27 | 66299 | unclassified *Myoviridae* | NC_026586 |
| pp vB_PaeM_PAO1_Ab29 | 66326 | unclassified *Myoviridae* | LN610588 |
| pp JG024 | 66275 | unclassified *Myoviridae* | NC_017674 |
| pp DL68 | 66111 | unclassified *Myoviridae* | KR054033 |
| S12-1 | 66257 | unclassified *Myoviridae* | LC102730 |
| R18 | 63560 | unclassified *Myoviridae* | LC102729 |
