# Supplementary material for: The use of informativity in the development of robust viromics-based examinations
Source: PeerJ. 2017 May 2;5:e3281. doi: 10.7717/peerj.3281 (PMC5417064; doi:10.7717/peerj.3281)
Supplement: Table S3 — SRA datasets included in the proof-of-concept investigation for Pseudomonas phage PB1 viruses. Each row listed was considered an individual sample. [file peerj-05-3281-s005.docx]

**Table S3.** SRA datasets included in the proof-of-concept investigation for Pseudomonas phage PB1 viruses. Each row listed was considered an individual sample.

| **Virome** | **SRA run datasets** |
| --- | --- |
| **I** | SRR1301999 |
|  | SRR1302020 |
|  | SRR1302010 |
|  | SRR1296481 |
|  | SRR1974493 |
|  | SRR1974494 |
|  | SRR1974490 |
|  | SRR1974491 |
|  | SRR1974495 |
|  | SRR1974496 |
|  | SRR1974497 |
|  | SRR1974498 |
|  | SRR1915829 |
|  | SRR1915851 |
|  | SRR1974488 |
|  | SRR1974489 |
|  | SRR1974499 |
|  | SRR1974500 |
|  | SRR1974501 |
|  | SRR1974502 |
|  | SRR1974503 |
|  | SRR1974504 |
|  | SRR1974505 |
|  | SRR1974506 |
|  | SRR1974507 |
|  | SRR1974508 |
|  | SRR1974509 |
|  | SRR1974510 |
|  | SRR1974511 |
|  | SRR1974512 |
|  | SRR1974513 |
|  | SRR1974514 |
|  | SRR1974515 |
|  | SRR1974516 |
|  | SRR1974517 |

| **Virome** | **SRA run datasets** |
| --- | --- |
| **II** | ERR019477 |
|  | ERR019478 |
| **III** | SRR001047 |
|  | SRR001075 |
|  | SRR001076 |
| **IV** | SRR013515, SRR013516, SRR013517 |
|  | SRR013520, SRR013521 |
| **V** | SRR014584 |
|  | SRR014585 |
|  | SRR014586 |
|  | SRR014587 |
|  | SRR014588 |
|  | SRR014589 |
| **VI** | SRR138365 |
|  | SRR155589 |
|  | SRR171296 |
| **VII** | SRR371574 |
|  | SRR648311 |
|  | SRR648312 |
|  | SRR648313 |
|  | SRR648314 |
